# Role of endothelial miR-24 in COVID-19 cerebrovascular events

**DOI:** 10.1186/s13054-021-03731-1

**Published:** 2021-08-25

**Authors:** Jessica Gambardella, Antonietta Coppola, Raffaele Izzo, Giuseppe Fiorentino, Bruno Trimarco, Gaetano Santulli

**Affiliations:** 1 Departments of Medicine (Cardiology) and Molecular Pharmacology, Wilf Family Cardiovascular Research Institute, Einstein Institute for Aging Research, Einstein-Sinai Diabetes Research Center , Albert Einstein College of Medicine, New York, NY USA; 2 International Translational Research and Medical Education (ITME) Consortium, Department of Advanced Biomedical Sciences , “Federico II” University, Naples, Italy; 3 COVID-19 Division, Azienda Ospedaliera di Rilevo Nazionale (AORN) “Ospedali dei Colli”, Naples, Italy

Levels of microRNAs (miRNAs) within extracellular vesicles (EVs) have been shown to be useful diagnostic and prognostic biomarkers in a number of disease states [1–3]. However, EVs miRNAs have never been investigated in COVID-19.

We recently demonstrated that miR-24 is expressed in human brain endothelial cells (ECs) and targets Neuropilin-1 [4], a co-factor needed for SARS-CoV-2 internalization that has been linked to cerebrovascular (CBV) manifestations of COVID-19 [5]. Henceforth, we hypothesized an association between plasma levels of endothelial EV miR-24 and the onset of CBV events in patients hospitalized for COVID-19. CBV events were defined by the presence of ischemic or hemorrhagic stroke (confirmed by imaging), migraine, or transient ischemic attack (no findings at imaging evaluation).

We obtained plasma from 369 patients hospitalized for COVID-19, consecutively enrolled from November 2020 to April 2021 at the “*Ospedali dei Colli*”. We excluded 48 patients with a history of CBV disease, cancer, atrial fibrillation, deep vein thrombosis, or unavailability of admission blood samples; thus, the study was conducted in 321 subjects. As a control age- and sex-matched COVID-19 negative population, we obtained plasma from 57 healthy donors and 37 patients with CBV disorders. A SARS-CoV-2 test (RT-qPCR) was performed in all subjects to confirm or rule out the COVID-19 diagnosis. EC-EVs were extracted from the plasma collected from these patients via serial centrifugation and CD31^+^ magnetic isolation [1], and EC-EVs miR-24 levels were quantified as described [1, 4, 6].

Clinical parameters of our population are reported in Table 1. CBV events were diagnosed in 58 COVID-19 patients. No significant differences in comorbidities and in therapeutic management were observed. We found that EC-EV miR-24 levels were significantly reduced in patients with *vs* without CBV disorders among COVID-19 patients, but not when examining subjects without COVID-19 (Table 1). These results were confirmed when subdividing our population according to the presence of ischemic or hemorrhagic findings at imaging evaluation (Fig. 1). Strikingly, using a stepwise multiple regression analysis, adjusting for age, hypertension, dyslipidemia, diabetes, and D-dimer, the association between EC-EV miR-24 and CBV disease in COVID-19 patients was confirmed [Wald: 17.723; Exp(B): 0.955, C.I. 95%: 0.935–0.976, *P* < 0.001].

**Table 1:** Main characteristics of our population

	COVID-19 negative			COVID-19 positive		
	NO CBV	CBV	*P*	NO CBV	CBV	*P*
	(57)	(37)		(263)	(58)	
Age (years)	59.4 ± 14.78	65.37 ± 12.75*	*0.04*	61.5 ± 14.2	63.6 ± 14.6	*0.292*
Sex (male, %)	50.9	59.5	*0.42*	54.7	55.1	*0.954*
BMI (kg/m^2^)	25.62 ± 3.83	25.44 ± 2.9	*0.8*	24.93 ± 3.59	25.01 ± 2.9	*0.865*
SBP (mmHg)	133.88 ± 16.1	143.08 ± 19.3*	*0.014*	137.76 ± 19.5	142.2 ± 19.3	*0.114*
DBP (mmHg)	79.93 ± 9.5	84.38 ± 8.8*	*0.025*	84.46 ± 9.47	86.3 ± 14.9	*0.239*
Hypertension (%)	26.3	51.3*	*0.013*	39.9	44.8	*0.493*
Glycemia (mg/dl)	104.9 ± 22.2	112.2 ± 27.8	*0.163*	109.39 ± 28.2	112.48 ± 42.7	*0.497*
Diabetes (%)	7.0	21.6*	*0.039*	12.5	17.2	*0.344*
Dyslipidemia (%)	24.5	43.2	*0.059*	30.8	34.4	*0.586*
Smoking (current/past, %)	14/22.8	21.6/37.8*	*0.032*	18.2/26.6	12.1/34.4^#^	*0.081*
D-dimer (µg/ml)	2.35 ± 1.73	3.52 ± 0.95*	*0.001*	2.80 ± 1.68	3.18 ± 1.83	*0.120*
IL-6 (pg/ml)	1.7 ± 1.1	4.0 ± 2.8*	*0.002*	7.5 ± 4.0^#^	8.4 ± 5.5^#^	*0.121*
TNFα (pg/ml)	4.5 ± 2.3	6.0 ± 4.7*	*0.035*	6.5 ± 4.2	5.8 ± 4.7	*0.271*
hs-CRP (µg/ml)	2.15 ± 1.1	2.6 ± 1.16	*0.07*	3.6 ± 3.2^#^	4.2 ± 2.9^#^	*0.144*
EC-EV miR-24 (copies/10 nl)	30.5 ± 14.6	29.85 ± 15.5	*0.827*	26.64 ± 20.9^#^	15.41 ± 14.7*^,#^	*0.001*

All *P* values in the table are reported in italic

Data on quantitative parameters are expressed as mean ± standard deviation; data on qualitative characteristics are expressed as percentage values or absolute numbers. BMI: Body mass index; CBV: cerebrovascular (events); DBP: diastolic blood pressure; EC-EV miR-24: level of miR-24 shuttled by endothelial (CD31^+^) extracellular vesicles; hs-CRP: high-sensitivity C-reactive protein; IL-6: interleukin-6; SBP: systolic blood pressure; and TNFα: tumor necrosis factor α. Following verification of normality (Shapiro–Wilk’s test) and equal variance (Bartlett’s test), continuous variables were compared using ANOVA followed by Tukey–Kramer test for independent samples, whereas variables not normally distributed were compared via the Kruskal–Wallis test; categorical data were compared using the χ^2^ test; *P < 0.05 versus NO CBV; ^#^*P* < 0.05 versus COVID-19 negative

**Fig. 1:**
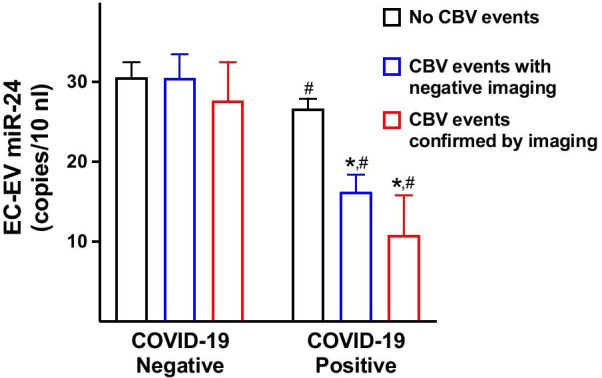
miR-24 levels were measured within endothelial extracellular vesicles (EC-EV), identified by the endothelial marker CD31. Cerebrovascular events (CBV) were divided in events with no findings at imaging evaluation, which included transient ischemic attacks (TIA) and migraine (*blue bars*), and ischemic or hemorrhagic stroke confirmed by imaging (*red bars*). Data are represented as mean ± SE; *P < 0.05 versus NO CBV; ^#^*P* < 0.05 versus COVID-19 Negative

To our knowledge, this is the first study showing an association between EC-EV non-coding RNA and clinical outcome in COVID-19 patients.

The main limitation of the present study is the relatively small size of our population; moreover, our findings, which are limited to Caucasian individuals, refer to subjects that have been hospitalized for COVID-19 and therefore cannot be generalized to patients with a mild disease.

We identified a significant association linking EC-EV miR-24 and CBV disorders, which could be valuable to understand the mechanisms underlying the pathophysiology of CBV complications in COVID-19. Indeed, low levels of EC-EV miR-24 suggest an increased expression of Neuropilin-1 in ECs [4]. Further analyses in larger groups are warranted to ratify our results, confirm their prognostic value, and investigate the role of miR-24 in other COVID-19-related neurologic events.

## Data Availability

The datasets used and/or analyzed during the current study are available from the corresponding author on reasonable request.
